# Effects of Ramadan fasting on aspirin resistance in type 2 diabetic patients

**DOI:** 10.1371/journal.pone.0192590

**Published:** 2018-03-12

**Authors:** Wahid Bouida, Kaouthar Beltaief, Houda Baccouche, Mouna Sassi, Zohra Dridi, Imen Trabelsi, Kamel Laaouiti, Taher Chakroun, Ilhem Hellara, Riadh Boukef, Nabil Sakly, Mohsen Hassine, Faouzi Added, Rabie Razgallah, Fadhel Najjar, Semir Nouira, On behalf of the Ramadan Research Group

**Affiliations:** 1 Emergency Department, FattoumaBourguiba University Hospital, Monastir, Tunisia; 2 Research Laboratory (LR12SP18) University of Monastir, Monastir, Tunisia; 3 Biological Laboratory, Maternity and Neonatal Medicine Center, Monastir, Tunisia; 4 Cardiology Department, FattoumaBourguiba University Hospital, Monastir, Tunisia; 5 Regional blood transfusion center, FarhatHached University Hospital, Sousse, Tunisia; 6 Hematology Department, FattoumaBourguiba University Hospital, Monastir, Tunisia; 7 Emergency Department, Sahloul University Hospital, Sousse, Tunisia; 8 Laboratory of Immunology, FattoumaBourguiba University Hospital, Monastir Tunisia; 9 Cardiology Department, AbderrahmanMami University Hospital, Ariana Tunisia; 10 Medis Laboratories, 1053, Tunis, Tunisia; 11 Biochemistry Department, FattoumaBourguiba University Hospital, Monastir Tunisia; Mexican Social Security Institute, MEXICO

## Abstract

**Aims:**

Ramadan fasting (RF) may affect aspirin resistance. We conducted this study in patients with cardiovascular risk (CVR) factors to assess the effect of RF on aspirin resistance and explore whether type 2 diabetes mellitus (DM) would influence this effect.

**Methods:**

A total of 177 stable patients with ≥2 CVR factors were recruited. All patients observed RF and were taking aspirin. Physical exam and standard biological tests including glycaemia and serum lipids data were performed before Ramadan (Pre-R), at the last week of Ramadan (R) and four weeks after the end of Ramadan (Post-R). In the same visits caloric intake was calculated and platelet reactivity to aspirin was assessed using Verify Now point-of-care assay.

**Results:**

In the overall population, there was no significant change in absolute aspirin reaction unit (ARU) values and in metabolic parameters. In DM patients (n = 127), ARU change from Pre-R values was+19.7 (p = 0.01) and +14.4 (p = 0.02) respectively at R and Post-R. During Ramadan, glycaemia, triglycerides, and cholesterol levels increased significantly and returned to Pre-R values thereafter. These changes were not observed in non-DM patients.

**Conclusions:**

During RF aspirin resistance increased only in DM patients. This effect persisted one month after Ramadan. Simultaneous alteration of glycemic control and increase of serum lipids levels could potentially be a favorable factor.

**Study registration:**

The protocol was registered at clinicaltrials.gov under: NCT02720133.

## Introduction

Ramadan fasting (RF)has been shown to be associated with metabolic disorders related to glycemic control and serum lipids levels [1–3]. It may also alter the effects of some pharmacologic agents resulting from the change in eating behavior and timing of medicines ‘taking[4–8].Anti-platelet agents such as aspirin are commonly used and their effect could be modified during RF. The potential effect of RF on aspirin resistance may be harmful[9,10] particularly in patients with type 2 diabetes mellitus (DM) known for their suboptimal response to anti-platelet agents[11–16]. Evaluating aspirin resistance during RF could have significant clinical relevance with regard to the management and monitoring of patients under antiaggregating agents while observing RF. This task became possible with the development of new simple assays to assess platelet reactivity. On predicting changes of aspirin resistance during RF in patients with cardiovascular disease we might prevent related adverse events [17]. Our aim was to evaluate the effect of RF on platelet reactivity in patients under aspirin treatment. In addition, we investigated whether this effect could be different between patients with and without DM.

## Patients and methods

### Participants

This prospective observational study included subjects with at least two cardiovascular risk factors according to Framingham classification[18].They were recruited from university and non-university medical centers. Participants were screened in outpatient clinics (cardiology, endocrinology, internal medicine, family medicine) when they presented for scheduled follow-up. Selection was based on the participant’s decision to fast, while taking aspirin therapy for at least six months (the daily dose was 100 mg). Exclusion criteria included age under 40 years, unstable diabetes, current or previous (14 days) use of glycoprotein IIb/IIIa or antidepressants, inability to give informed consent, baseline platelet count <100x10^6^/L, or terminal chronic disease. The study was approved by the Institutional Review Board of Fattouma Bourguiba University Hospital (25/03/2016)and all patients provided written informed consent. After screening, the study design and requirements were thoroughly explained to the participants. This trial was retrospectively registered at clinicaltrial.gov (number NCT02720133). Recruitment of patients in this study started before its registration because we did not know that we need to register the study before patient recruitment. Medis Laboratories had no role in study design, data collection, data analysis, data interpretation, or writing of the report. All authors had full access to all the data in the study and had final responsibility for the decision to submit for publication. We also confirm that all ongoing and related trials for this drug/intervention are registered.

### Methods

The study lasted four years (2010–2014) with three separate assessment visits in each year:1) the week before Ramadan which represented the baseline period (Pre-R); 2) the last week of Ramadan (R); 3) and during the last week of the month following Ramadan (Post-R). The duration of fasting was approximately 12 h from sunrise to sunset (the time of abstinence from food) during a 30 d period. Each patient served as his own control and was required to take the prescribed aspirin dose daily. The assessment in each of the three visits involved physical exam and blood sampling for standard biological tests. Body weight and height were performed and body mass index (BMI) was calculated as body weight (kg) divided by squared height in meters (m^2^). Physical examination was carried out in all participants including systolic and diastolic blood pressure, and cardiac rate. The visit is completed by a questionnaire on diet beginning 2 days before the blood sampling. No special nutritional regimen was applied to the participants during the study. All subjects were encouraged to continue their usual lifestyle and activities. The rate of hypoglycemic (symptomatic and non-symptomatic) and hyperglycemic episodes requiring ED admission was recorded within the three periods of the study. Hypoglycemia was defined as blood glucose < 3.5 mmol/L. Compliance to current treatment (aspirin, clopidogrel, oral hypoglycemic agents, statins…) was assessed by the attending physician based on interview and pill count. Venous blood samples were collected from the enrolled participants during the three time points. The time of blood sampling in the study was 9–10 am, at which all participants were fast. And for the purpose of the study, patients were asked to take their last meal (“sehour”) between 11 o’clock pm and midnight. Blood samples were analyzed directly for hemoglobin, hematocrit, and platelet cell count.Platelet reactivity was assessed by the Verify Now point-of-care assay (Accumetrics, San Diego, CA, USA) using venous blood samples collected in tubes containing 3.2% sodium citrate. Results are reported as aspirin reaction units (ARU) and aspirin resistance was defined as ARU >550. ARU results were not revealed to patients and their primary physicians. Blood biochemical measurements included glucose, total cholesterol (TC) and triglycerides (TG), and high-density lipoprotein cholesterol (HDL-C) concentrations. Low-density lipoprotein cholesterol (LDL-C) was calculated using the Friedewald formula: [LDL-chol] = [total chol]—[HDL-chol]—([TG] /2.2).

### Statistical analysis

The categorical data are presented as the percentage frequency of occurrence. All continuous data are presented as either the median with 95% confidence interval (CI) or the mean with SD according to the distribution of the data. The Kolmogorov-Smirnov test was performed to assess the normal distribution. Each subject served his own control by comparing his/her values before Ramadan with those during and after Ramadan. Differences between results were analyzed using paired samples t test for normally distributed parameters and Wilcoxon signed Rank test for not normally distributed parameters. Comparison was performed between patients with and without DM. Statistical significance was considered at p<0.05 for all tests. Statistical analyses were conducted by using SPSS statistical software (version 11.5, SPSS Inc. Chicago, IL).

## Results

**Fig 1:**
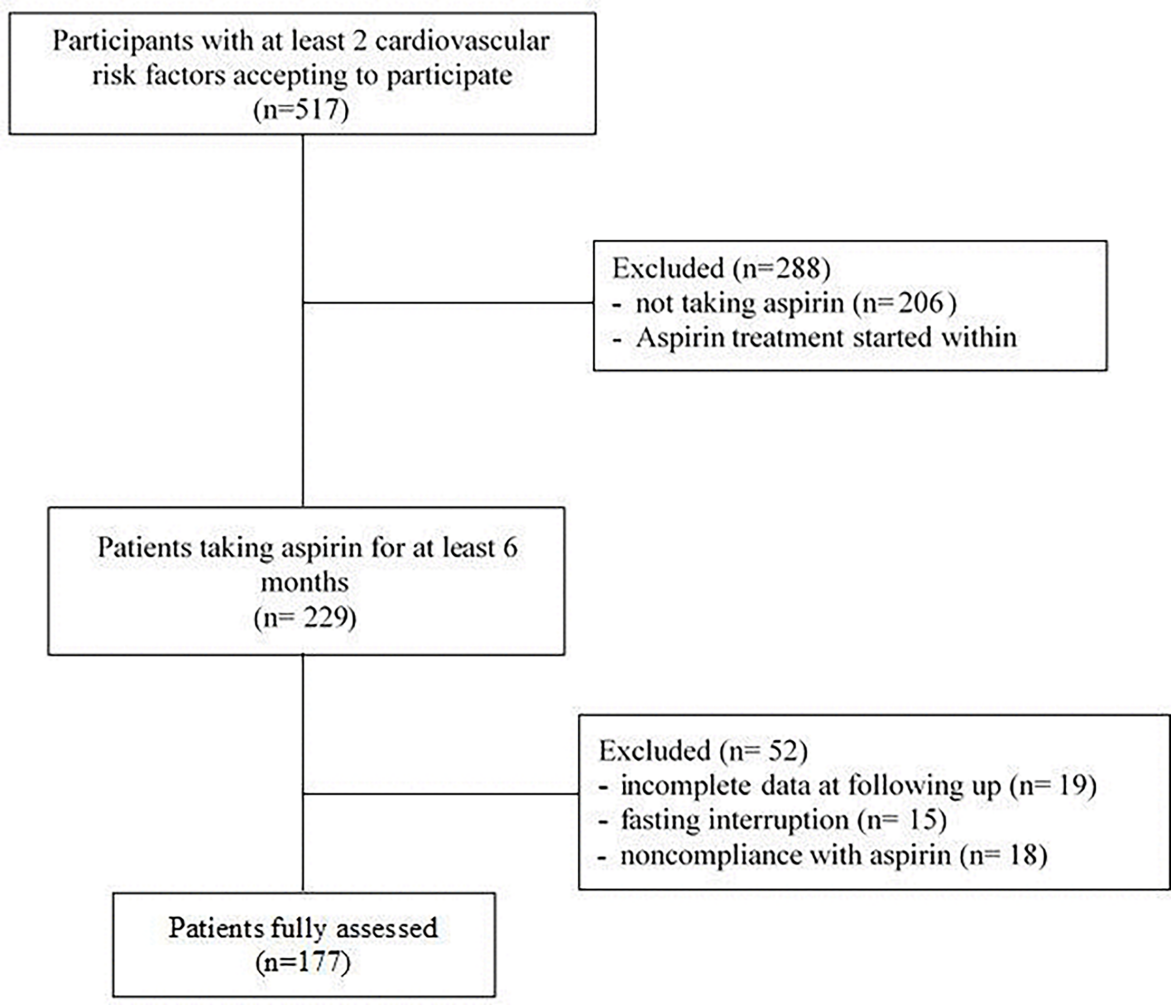
Study profile.

Of the 517 participants screened, 206 not taking aspirin were excluded. From the 311 patients under aspirin 82(26.3%) were excluded because aspirin treatment was started within the six last months before this study. From the remaining patients we excluded from the analysis 19 patients for incomplete data at follow-up, 15 patients because they stopped fasting, and18 patients for noncompliance with aspirin treatment. Overall177 participants were included and studied at the three planned visits (Fig 1).

Demographic and clinical characteristics of the participants are summarized in Table 1. The mean age was 60.0±10.2 years and 66.1% were men and 127 (71.7%) had a type diabetes mellitus (DM). Concomitant treatment with clopidogrelwasreportedin60 patients (33.9%).

**Table 1:** Baseline characteristics.

	Total n = 177
Age years (mean ± SD)	60 ± 10.19
Male gender; n (%)	117 (66.1)
Dyslipidemia n (%)	121 (68.4)
Diabetes n (%)	127 (71.8)
HTA n (%)	133 (75.1)
Smoking n (%)	67 (37.9)
Coronary artery disease n (%)	78 (44.1)
**Number of CAD**	
2	39 (22)
3	61 (34.5)
≥4	59 (33.3)
**Treatment**	
Aspirin n (%)	177 (100)
Statins	96 (54.2)
Oral antidibetics	94 (53.1)
Angiotensin converting enzyme inhibitors	99 (55.9)
Beta-blokers	45 (25.4)
Diuretics	35(19.8)
Angiotensin receptor antagonists	23 (13)
Vitamin K antagonists	2 (1.1)

Blood pressure and cardiac rate did not change significantly between the three periods. Caloric intake decreased slightly during RF and increased thereafter. All these changes were not significant as was the distribution of caloric intake between glucids, lipids and proteins. BMI and weight decreased significantly during RF and returned to Post-R values (Table 2).

**Table 2:** Clinical and caloric intake changes during the three protocol periods.

	Pre-Ramadan Mean (SD)	Ramadan Mean (SD)	Post-Ramadan Mean (SD)
Systolic arterial pressure (mmHg)	144.6 (24.2)	143.5 (26.47)	140.6 (25.9)
Diastolic arterial pressure (mmHg)	84.1 (13.8)	81.6 (16.1)	79.3 (13.3)
Pulse (b/min)	72.6 (12.49)	72.4 (11.49)	75.1 (12.2)^£^
Weight (Kg)	82.1 (14.1)	82.4(15.4)	81.2(14)
Body mass index (Kg/m^2^)	30.9 (5.5)	31.1 (6.0)	30.8 (6.6)
Caloric total intake (kcal/j)	1721(462)	1520(1545)	1951(1670)^£^
Carbohydrate (%)	55.6 (7.0)	55.07 (8.2)	55.3 (8.5)
Protein (%)	16.5 (4.1)	18.1 (3.7)*	16.5 (4.5)^£^
Fat (%)	27.7 (7.2)	26.7 (8.3)	28 (7.8)

*p<0.05 between pre-Ramadan and Ramadan

^£^p<0.05 between Ramadan and post-Ramadan

Time intervals between aspirin intake and Verify Now testing were similar for the three visits. Results of aspirin resistance for the three study periods are presented in Table 3.

**Table 3:** Platelet reactivity and aspirin resistance in patients with and without diabetes mellitus.

	All	DM	Non DM
	n = 177	n = 127	n = 50
**Pre Ramadan**			
ARU mean (SD)	466.3 (86.3)	464.9 (89.5)	470 (78.5)
Aspirin resistance n (%) ARU>550	35 (20.9)	23 (18.1)	12 (24)
**Ramadan**			
ARU mean (SD)	476.7 (102)	484.5 (106.3)*	456.9 (88.07)^£^
Aspirin resistance n (%) ARU>550	47 (26.5)*	38 (29.9)*	9 (18)
**Post Ramadan**			
ARU mean (SD)	479.2 (90.7)	479.3(93.8)	479.1(83.4)
Aspirin resistance n (%) ARU>550	47 (26.5)*	33 (25.9)*	14 (28)

DM: diabetes mellitus

*p<0.05 compared to Pre-Ramadan

^£^p<0.05 compared to patients with DM.

**Fig 2:**
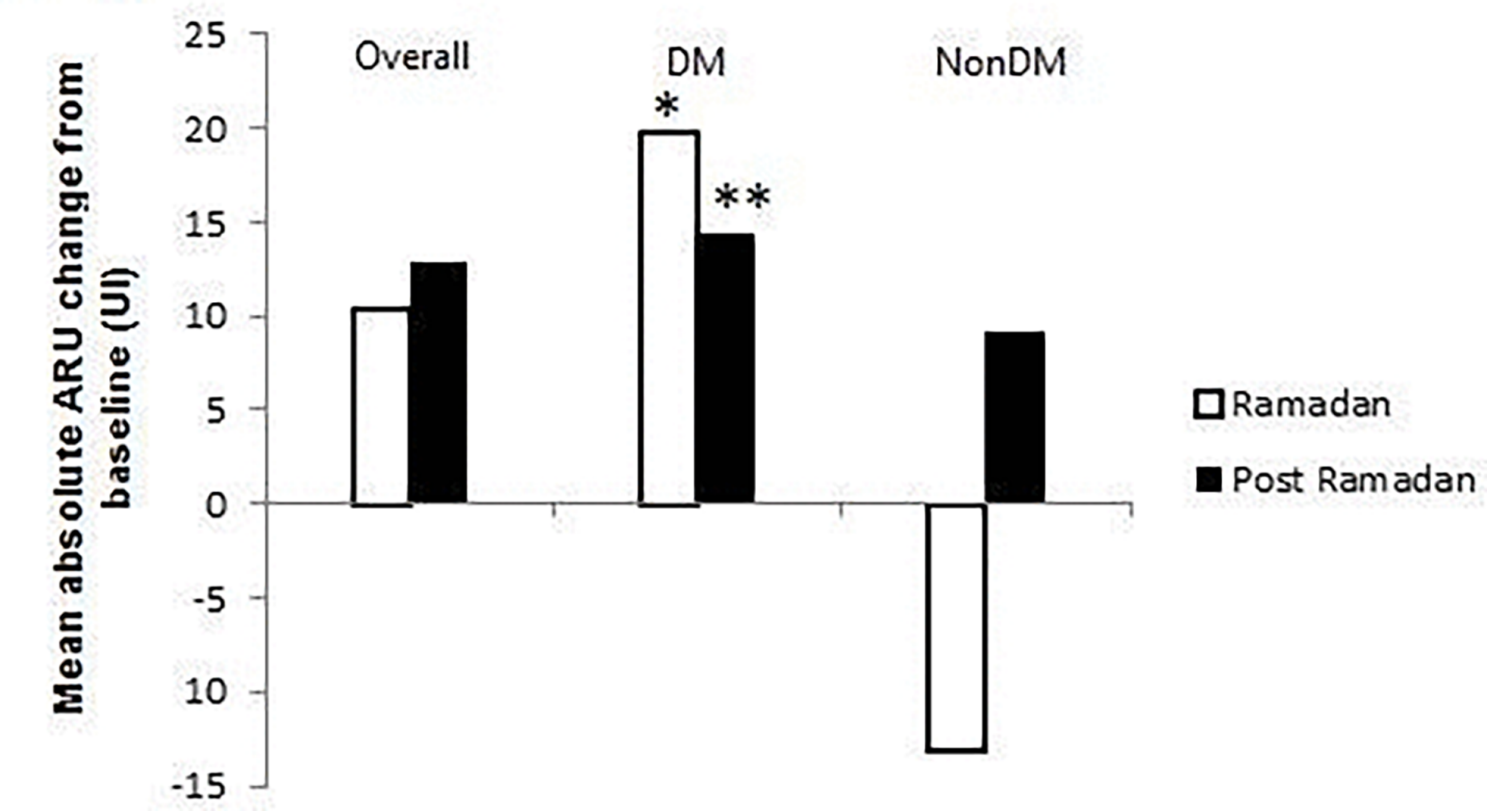
Mean absolute ARU change from baseline during and after Ramadan. *p<0.05 between Ramadan and Post-Ramadan.

ARU values did not change significantly between Pre-R, R and Post-R periods in the overall population (p = 0.03). In patients with DM, the absolute increase of ARU from baseline was +19.7 during RF (p = 0.02) and +14.4 at Post-R period (p = 0.05) (Fig 2). Conversely, in non DM participants ARU values decreased at Ramadan and increased at Post-Ramadan compared to baseline; however, these changes were not significantly different between the three periods. Aspirin resistance rate (ARU >550) did not change significantly between the three periods when we consider the overall population.

However, in patients with DM the rate of aspirin resistance increased significantly during Ramadan and in Post-Ramadan periods compared with Pre-R (respectively 29.9% and 26.0vs18.1%;(p = 0.001). With regard to standard biologic tests we found that glycemia increased significantly during Ramadan compared to Pre-R (9.5±4.5mmol/Lvs 8.9±4.5 mmol/L; p = 0.04) and returned to Pre-R values after Ramadan (8.6±4.1mmol/L; p = 0.23). Serum TG levels also increased significantly from 1.58±0.77 mmol/L at pre-R period to1.94±0.84mmol/Lat Ramadan period (p<0.001) and decreased to 1.65±0.92 at post-R period (p = 0.18) (Table 3). Cholesterol values demonstrated similar changes as triglycerides (Table 4). HDL cholesterol decreased from 1.17±0.32mmol/L at pre-R period to 1.09±0.30mmol/L (p = 0.02) during Ramadan, and returned to baseline values at post Ramadan period (1.22±0.20 mmol/L). LDL cholesterol did not change significantly between the three periods (Table 4). The same metabolic changes were observed in patients with DM but not in patients without DM.

**Table 4:** Biological changes during the three protocol periods.

	All			Patients with DM n = 127			Patients without DM n = 50		
	Pre-Ramadan mean (SD)	Ramadan mean (SD)	Post-Ramadan mean (SD)	Pre-Ramadan mean (SD)	Ramadan mean (SD)	Post-Ramadan mean (SD)	Pre-Ramadan mean (SD)	Ramadan mean (SD)	Post-Ramadan mean (SD)
**Hematological**									
**Hemoglobin (g/dl)**	13.08(1.35)	3.1 (1.3)	13.1(1.2)	13 (1.29)	13.05 (1.4)	13.1 (1.2)	3.2 (1.5)	13.2 (1.3)	13.2 (1.5)
**Hematocrite (%)**	41.1 (4.3)	39.4 (4.05)*	38.9(3.6)^`^	40.9 (4.23)	39.1 (3.9)*	38.8 (3.3)^§^	41.58 (4.4)	40.2(4.1)	39.2 (4.3)^§^
**Platelets count (x10**^**3**^**/ml)**	238.2(67.5)	237.7(63.8)	234.6(66.2)	239.5(69.1)	237.1 (64.6)	233.8 (68.2)	235(63.8)	239(62.3)	236.5(61.3)
**Biochemical**									
**Glycemia (mmol/l)**	8.9 (4.5)	9.5(4.37)	8.64 (4.12)	10.1 (4.6)	0.7 (4.5)	9.7(4.3)^`^	5.9 (2.5)	6.3 (1.3)	5.8 (1.3)^£^
**Cholesterol (mmol/l)**	4.36 (1.18)	4.4 (1.28)*	4.3 (1.22)^£^	4.2 (1.12)	4.4 (1.2)*	4.2 (1.22)^£^	4.6 (1.2)	4.7 (1.2)	4.6 (1.15)
**Triglycerides (mmol/l)**	1.58 (0.77)	1.94 (1.3)*	1.35 (0.9)^£^	1.6 (0.79)	2 (1.4)*	1.6 (0.93)^£^	1.5 (0.7)	1.7 (0.9)	1.6 (0.93)
**LDL cholesterol (mmol/l)**	2.47(0.99)	2.5 (1.06)	2.2 (3.25)	2.34(0.93)	2.38(1.01)	2 (3.7) ^£^	2.8 (1.07)	2.8 (1.12)	2.7 (0.96)
**HDL cholesterol (mmol/l)**	1.16 (0.32)	1.09 (0.3)*	1.39 (3.01)	1.16(0.31)	1.07(0.28)*	1.46(3.5)	1.17(0.3)^§^	1.12 (0.3)*	1.22 (0.3)^£^

DM: diabete mellitus

*p<0.05 between Pre-Ramadan and Ramadan

^£^p<0.05 between Ramadan and Post-Ramadan

^§^p<0.05 between Pre-Ramadan and Post-Ramadan

LDL/HDL: low-density/high-density lipoprotein

## Discussion

We demonstrated that RF significantly increased aspirin resistance only in patients with DM and persisted weeks later. In patients without DM, no significant changes in aspirin resistance were observed. In addition, we showed that these effects were associated with a significant increase of glycemia and lipids levels including serum TG and cholesterol.

A number of clinical studies have correlated aspirin resistance with long term adverse clinical events not only in patients with coronary artery disease but also in persons with ischemic stroke or peripheral arterial disease[14–16].However, this finding was not observed in other studies [19]. Data on the frequency of aspirin resistance varied greatly[11–13], largely because of the differences in the definition of resistance and the laboratory method used. Although the concept of aspirin resistance has existed for more than 30 years, no previous study has investigated the potential influence of RF on aspirin resistance and especially in diabetic patients[20, 21].RF could significantly modify the response to aspirin through multiple factors including changes in glycaemia and serum lipid levels. Lifestyle disturbance during RF and the consequent psychical stress may increase catecholamine’s concentration that also leads to higher platelets reactivity[22].Although participants in the present study were encouraged to continue their normal lifestyle, it is difficult to maintain exactly the same activities in Ramadan month; in this point we totally agree with the reviewer. Consequently, we cannot exclude the involvement of other factors in aspirin resistance during our study. In diabetic individuals this risk is more important which explain their predisposition for aspirin resistance [20, 21].Metabolic disturbances related to RF in particular in DM patients are probably the leading mechanism to the increased aspirin resistance[23, 24].Major glycemic excursions observed during RF may lead to non-enzymatic glycosylation of platelet membrane proteins and may change their structure and function[25,26]. High glycemic levels may also affect platelet aspirin reactivity through an increase of superoxide production or inflammatory mediators release[21,27]. It was demonstrated that inflammatory markers correlated with response to aspirin and clopidogrel dual therapy, and in the same time, hyperglycemia positively correlated with increased thrombus formation[27].In the present study, we found that higher ARU values in the fasting period was associated with a significant increase of serum triglycerides and cholesterol which suggest that RF may have a lipid-related prothrombotic action. To our knowledge this is the first study where it was attempted to assess the effects of fasting on aspirin resistance during Ramadan. Millions of Muslims under aspirin treatment observe fasting contrasting with a lack of evidence based guidance in this issue. As optimal anti-platelet inhibition is essential in DM patients with CAD, and according to our results, we believe that these patients should be considered at increased risk of aspirin resistance during and after RF and should be closely managed. Reinforcing the dose of current medications (statins, oral antidiabetics) or switching to more potent antiplatelet agents would be beneficial.

### Study limitations

First, although we attempted to verify compliance to aspirin and the treatment regimens during the three study periods, we cannot absolutely rule out inadequate compliance. Second, platelet function was assessed only by one method which is the VerifyNow assay. It should be highlighted in this issue that this method is one of the most widely accepted tests of platelet function [17].Third, although we did not observe any thrombotic events during the study period we must recognize that this study was not designed to evaluate clinical outcome; it is not in the scope of our work to assess the clinical relevance of our results. We need specific clinical studies to assess whether the increase of aspirin resistance correlates with thrombotic events in diabetic patients during RF. Finally, the observed effect was produced by a particular model of Ramadan fasting and that it is not necessarily observed with any other Ramadan model.

## Conclusion

Our study found that RF is associated with an increase of aspirin resistance only in patients with DM. Alteration of glycemic control and serum lipid balance in these patients are the potential factors leading to aspirin resistance increase. Patients with DM should be closely monitored with serial diagnostic testing of platelet function during RF.

## Supporting information

S1 File Supporting file 1. CRF English.
(DOCX)

S2 File Supporting file 2. Consent of the patient.
(DOC)

S3 File Supporting file 3. CONSORT 2010 Flow Diagram (1).doc.
(DOC)

S4 File Supporting file4. CONSORT 2010 Checklist.doc.
(DOC)
