# Expression of Concern: Effects of Ramadan fasting on aspirin resistance in type 2 diabetic patients

**DOI:** 10.1371/journal.pone.0342146

**Published:** 2026-02-03

**Authors:** The PLOS One Editors

After publication of this article [1], concerns were raised about the ethics approvals, patient consent, and text overlap. Specifically:

- The Methods state that the study lasted four years between 2010 and 2014; however, the reported information suggests that the approval of the Institutional Review Board of Fattouma Bourguiba University Hospital was issued in 2016.
- The Methods states that subjects were recruited from university and non-university medical centers but refers to approval from the Institutional Review Board of Fattouma Bourguiba University Hospital only.
- The patient consent form in S2 File appears to relate to a different study.
- There is text overlap between the Methods and Discussion sections of [1] and [2], and [2] was not cited in [1].

The corresponding author stated that the Institutional Review Board of Fattouma Bourguiba University Hospital initially approved a Ramadan fasting study with an open objective to assess the clinical and biological changes during Ramadan month, and that the date of 25^th^ March 2016 refers to a secondary IRB approval specifically for the aspirin resistance study in [1]. They also stated that the incorrect patient consent form in S2 File was included in error and that only Fattouma Bourguiba University Hospital was involved in the ethical approval process for [1]. The corresponding author noted that the following are no longer available:

- The original data underlying [1]
- The correct patient consent form for [1]
- The original and secondary ethics approvals for [1]

PLOS requested institutional follow-up. At the time of publication of this Expression of Concern, the institution has not provided input, and therefore the above questions about whether the study met with appropriate clinical ethical standards are unresolved.

Regarding the text overlap between [1] and [2], the corresponding author stated that both studies are on prospective Ramadan cohorts with type 2 diabetics and cardiovascular risk, with one on aspirin and the other on clopidogrel. They stated that each article has nearly identical design, but the patient cohorts and outcomes differ and each paper reports independent sample sizes and endpoints.

In following up on these issues, a statistical reviewer noted that:

- The subgroup analysis performed did not have sufficient power and was performed without evidence of effect modification.
- The use of ‘effects’ in an observational study and the lack of a control group may increase risk of bias.
- There are inconsistencies between the methodology reported in the Statistical analysis section for presentation of continuous data (stated as either the median with 95% confidence interval (CI) or the mean with SD according to the distribution of the data) and the subsequent presentation of the results.
- The reporting of the methodology is incomplete.

Contrary to the declaration in the Data Availability statement, the original raw data files supporting the article’s results were not provided with the article, and the authors have not provided these data following editorial request. As the original raw data files supporting the article’s results have not been made available, this article [1] does not comply with the PLOS Data Availability policy.

In light of the above concerns, the *PLOS One* Editors issue this Expression of Concern.

The corresponding author provided additional clarifications and corrections as follows:

- Non-parametric tests (e.g., Wilcoxon signed-rank test) were used for within-group comparisons and Mann-Whitney U tests for between-group comparisons when variables were non-normally distributed, and t-tests otherwise. For repeated measures across time points, Friedman test or repeated measures ANOVA was applied depending on distribution. The comparison between patients with and without diabetes mellitus (DM) was performed using Mann-Whitney U tests for continuous variables (due to non-normality) and chi-square/Fisher’s exact tests for categorical variables.
- Table 3 presents absolute ARU values stratified by DM status at each time point, not difference-in-difference values. The subgroup analysis aimed to explore possible differences in platelet response during Ramadan fasting between diabetic and non-diabetic participants, but no formal interaction testing was performed.
- Diabetes status was collected at baseline by combining patient self-report with confirmation from medical records including fasting blood glucose, HbA1c, and treatment history.
- The 14^th^ sentence of the Results section is incorrect. The correct sentence is: ARU values decreased significantly between the Pre-Ramadan and Ramadan periods (p = 0.03), and between the Pre-Ramadan and Post-Ramadan periods (p = 0.03) in the overall population.
- There is an error in the Table citation in the 20^th^ sentence of the Results section. The correct sentence is: Serum TG levels also increased significantly from 1.58 ± 0.77 mmol/L at pre-R period to 1.94 ± 0.84 mmol/L at Ramadan period (p < 0.001) and decreased to 1.65 ± 0.92 at post-R period (p = 0.18) (Table 4).
- The caption of Fig 2 is incorrect. Please see the correct caption below.

**Fig 2:**
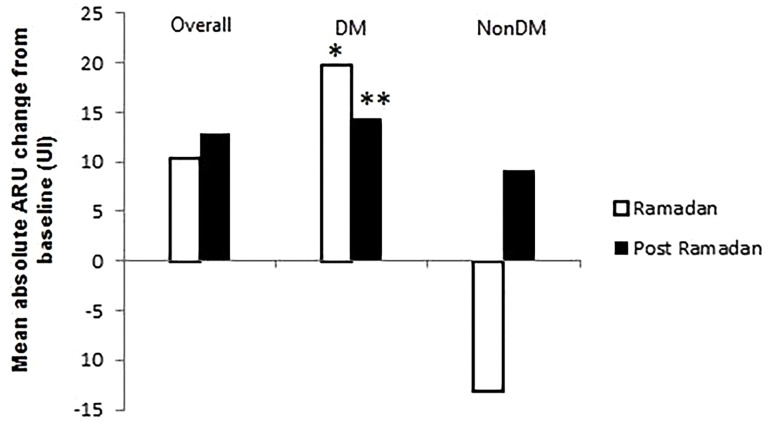
Mean absolute ARU change from baseline during and after Ramadan. *p < 0.05 between Ramadan and Post-Ramadan. **p < 0.05 between Pre-Ramadan and Post-Ramadan.
