# Supplementary material for: Effects of Ramadan fasting on aspirin resistance in type 2 diabetic patients
Source: PLoS One. 2018 Mar 12;13(3):e0192590. doi: 10.1371/journal.pone.0192590 (PMC5846719; doi:10.1371/journal.pone.0192590)
Supplement: S1 File — CRF English. (DOCX) [file pone.0192590.s001.docx]

**Effets Effects of Ramadan fasting on aspirin resistance in type 2 diabetic patients .**

***Ramadan Research Group***

**PATIENT CRF**

**Name**

**____________________________________________________**

**card number**

**__________________**

**Sent by**

**__________________**

**PHONE**

**1 : __ __ __ __ __ __ __ __**

**2 : __ __ __ __ __ __ __ __**

**Date of 1st consultation: __ __ | __ __ | 201 __**

**Year of birth : __ __ __ __ Sexe :** Homme 🞎 Femme 🞎

**Profession :** ………………………………………………………….

**Adress :** ………………………………………………………….

**Made the Jeun :** yes🞎 no🞎

**CARDIOVASCULAR RISK FACTORS:**

1. HTA : 🞎 Seniority : __ __ ans
2. Diabete : 🞎 Seniority : __ __ ans
3. Dyslipidemia : 🞎
4. smoking  : 🞎
5. Coronary heredity: 🞎

**ANTECEDENTS**

1. Heart failure : 🞎 NYHA : __ __
2. Revascularization procedure
   1. ATC : 🞎 Seniority  : __ __ ans
   2. PAC  : 🞎 Seniority  : __ __ ans
3. others  : 🞎 ………………………………………………………………………………………………………………………………………………………………………

**SPECIFIC TREATMENTS**

| **Médicament** |  | **Daily Dose** | **Time of the last take** | **Date of first take** |
| --- | --- | --- | --- | --- |
|  |  |  |  |  |
| **Aspirine** |  | **__ __ __ __** | **__ __ h : __ __ min** | **__ __ \| __ __ \| 201 __** |
| **Clopidogrel** |  | **__ __ __ __** | **__ __ h : __ __ min** | **__ __ \| __ __ \| 201 __** |
| **Oestrogène** |  | **__ __ __ __** | **__ __ h : __ __ min** | **__ __ \| __ __ \| 201 __** |
| **AVK** |  | **__ __ __ __** | **__ __ h : __ __ min** | **__ __ \| __ __ \| 201 __** |
| **Vit K** |  | **__ __ __ __** | **__ __ h : __ __ min** | **__ __ \| __ __ \| 201 __** |
| **Autre anti ADP** |  | **__ __ __ __** | **__ __ h : __ __ min** | **__ __ \| __ __ \| 201 __** |
|  |  |  |  |  |
| **IPP ou antiacides**  **Précisez :** …………………………… |  | **__ __ __ __** | **__ __ h : __ __ min** | **__ __ \| __ __ \| 201 __** |
| **Statines** |  | **__ __ __ __** | **__ __ h : __ __ min** | **__ __ \| __ __ \| 201 __** |
|  |  |  |  |  |
| **other traitement :** ………………………………………………………………………………………………………………… | | | | |

**PHYSICAL EXAMINATION**

1. SAP/ DAP ___ ___ ___ | ___ ___ ___ mmHg pulse : ___ ___ ___ bpm
2. wheight : ___ ___ ___ kg length : ___ ___ cm BMI : ___ ___kg.m^-2^
3. Abdominal perimeter: ___ ___ cm

**Shour  hour:** **___ ___**

**Sampling time :** **___ ___**

**FOOD SURVEY**

| **Breakfast** | **Lunch** | **Dinner** |
| --- | --- | --- |
| **_________________ __ __ ___**  **_ __ __ ___________________**  **__ __ __ __________________**  **__ __ __ __________________**  **__ __ __ ____ __ __ ________**  **__ __ __ ____ __ __ ________**  **__ __ __ ____ __ __ ________**  **_________________ __ __ ___**  **_ __ __ ___________________**  **__ __ __ __________________**  **__ __ __ __________________**  **__ __ __ ____ __ __ _______** | **_________________ __ __ ___**  **_ __ __ ___________________**  **__ __ __ __________________**  **__ __ __ __________________**  **__ __ __ ____ __ __ ________**  **__ __ __ ____ __ __ ________**  **__ __ __ ____ __ __ ________**  **_________________ __ __ ___**  **_ __ __ ___________________**  **__ __ __ __________________**  **__ __ __ __________________**  **__ __ __ ____ __ __ ________** | **_________________ __ __ _______________________**  **_ __ __ _______________________**  **__ __ __ ______________________**  **__ __ __ ____________________**  **__ __ __ ____ __ __ ______________________**  **__ __ __ ____ __ __ _____________________**  **__ __ __ ____ __ __ _____** |

|  |  | | 1,2,3 times/ month | | | | | 1,2,3,4,5,6,7 times/ week | | | | | | | | | | | | |
| --- | --- | --- | --- | --- | --- | --- | --- | --- | --- | --- | --- | --- | --- | --- | --- | --- | --- | --- | --- | --- |
|  | never | | **1** | | **2** | **3** | | **1** | | **2** | **3** | | | **4** | | **5** | | **6** | | **7** |
| How much do you consume**?** |  | |  | |  |  | |  | |  |  | | |  | |  | |  | |  |
| • olives / olive oils • Soft drinks • fries • toast with butter • fruits (fresh / compotes / squeezed) • Cakes: modern / traditional • Carrots • Honey jam • Dates • Cucumber • Boiled egg • Brika • Spinach • Lettuce • Green cabbage • Cauliflower • Broccoli • Beans • Beets • Lawyers • offal • Liver • Grapefruit • Marshmallow |  | |  | |  |  | |  | |  |  | | |  | |  | |  | |  |
| How much time do you spend on? |  | |  | |  |  | |  | |  |  | | |  | |  | |  | |  |
| walking ride a bicycle gardening / DIY Housework Sport (swimming, football, aerobics, ..) Sit in front of the TV Sit in front of the computer |  | |  | |  |  | |  | |  |  | | |  | |  | |  | |  |
|  |  | |  | |  |  | |  | |  |  | | |  | |  | |  | |  |
|  |  | |  | |  |  | |  | |  |  | | |  | |  | |  | |  |
|  |  | |  | |  |  | |  | |  |  | | |  | |  | |  | |  |
|  |  | |  | |  |  | |  | |  |  | | |  | |  | |  | |  |
|  |  | |  | |  |  | |  | |  |  | | |  | |  | |  | |  |
|  |  | |  | |  |  | |  | |  |  | | |  | |  | |  | |  |
| How are you sleeping? | |  | | | | | | | | | | | | | | | | | | |
| daily sleep time | | | | .............. H.............. mn | | | | |  | | | |  | |  | | | |  | |
| • daily wake up time | | | | ............... H.............. mn | | | | |  | | | |  | |  | | | |  | |
| • daily nap time Do you feel excessively sleepy during the day? | | | | .............. H.............. mn | | | | |  | | | |  | |  | | | |  | |
| Do you feel excessively sleepy during the day? | | | | never | | | Rarely   | | | | | Regularly | | | | | Often   | | | |

**Date of 2^nd^ consultation: __ __ | __ __ | 201 __**

**PHYSICAL EXAMINATION**

1. SAP/ DAP ___ ___ ___ | ___ ___ ___ mmHg pulse : ___ ___ ___ bpm
2. wheight : ___ ___ ___ kg
3. Abdominal perimeter: ___ ___ cm

**THERAPEUTIC ADJUSTMENT**

**Time of the last take**

**Aspirine __ __ h : __ __ min**

**Clopidogrel __ __ h : __ __ min**

**AVK __ __ h : __ __ min**

**other traitements :** ……………………………………………………………………………………………………………………

**Shour time:** **___ ___**

**Sampling time:** **___ ___**

**FOOD SURVEY**

| **Shour** | **Dinner** |
| --- | --- |
| **_________________ __ __ ___**  **_ __ __ ___________________**  **__ __ __ __________________**  **__ __ __ __________________**  **__ __ __ ____ __ __ ________**  **__ __ __ ____ __ __ ________**  **__ __ __ ____ __ __ ________**  **_________________ __ __ ___**  **_ __ __ ___________________**  **__ __ __ __________________**  **__ __ __ __________________**  **__ __ __ ____ __ __ _______** | **_ __ __ ___________________**  **__ __ __ __________________**  **__ __ __ __________________**  **__ __ __ ____ __ __ ________**  **__ __ __ ____ __ __ ________**  **__ __ __ ____ __ __ ________**  **_________________ __ __ ___**  **_ __ __ ___________________**  **__ __ __ __________________**  **__ __ __ __________________**  **__ __ __ ____ __ __ _______** |

|  |  | | 1,2,3 times/ month | | | | | 1,2,3,4,5,6,7 times/ week | | | | | | | | | | | | |
| --- | --- | --- | --- | --- | --- | --- | --- | --- | --- | --- | --- | --- | --- | --- | --- | --- | --- | --- | --- | --- |
|  | never | | **1** | | **2** | **3** | | **1** | | **2** | **3** | | | **4** | | **5** | | **6** | | **7** |
| How much do you consume**?** |  | |  | |  |  | |  | |  |  | | |  | |  | |  | |  |
| • olives / olive oils • Soft drinks • fries • toast with butter • fruits (fresh / compotes / squeezed) • Cakes: modern / traditional • Carrots • Honey jam • Dates • Cucumber • Boiled egg • Brika • Spinach • Lettuce • Green cabbage • Cauliflower • Broccoli • Beans • Beets • Lawyers • offal • Liver • Grapefruit • Marshmallow |  | |  | |  |  | |  | |  |  | | |  | |  | |  | |  |
| How much time do you spend on? |  | |  | |  |  | |  | |  |  | | |  | |  | |  | |  |
| walking ride a bicycle gardening / DIY Housework Sport (swimming, football, aerobics, ..) Sit in front of the TV Sit in front of the computer |  | |  | |  |  | |  | |  |  | | |  | |  | |  | |  |
|  |  | |  | |  |  | |  | |  |  | | |  | |  | |  | |  |
|  |  | |  | |  |  | |  | |  |  | | |  | |  | |  | |  |
|  |  | |  | |  |  | |  | |  |  | | |  | |  | |  | |  |
|  |  | |  | |  |  | |  | |  |  | | |  | |  | |  | |  |
|  |  | |  | |  |  | |  | |  |  | | |  | |  | |  | |  |
|  |  | |  | |  |  | |  | |  |  | | |  | |  | |  | |  |
| How are you sleeping? | |  | | | | | | | | | | | | | | | | | | |
| daily sleep time | | | | .............. H.............. mn | | | | |  | | | |  | |  | | | |  | |
| • daily wake up time | | | | ............... H.............. mn | | | | |  | | | |  | |  | | | |  | |
| • daily nap time Do you feel excessively sleepy during the day? | | | | .............. H.............. mn | | | | |  | | | |  | |  | | | |  | |
| Do you feel excessively sleepy during the day? | | | | never | | | Rarely   | | | | | Regularly | | | | | Often   | | | |

**Date of 3^rd^ consultation: __ __ | __ __ | 201 __**

**PHYSICAL EXAMINATION**

1. SAP/ DAP ___ ___ ___ | ___ ___ ___ mmHg pulse : ___ ___ ___ bpm
2. wheight : ___ ___ ___ kg
3. Abdominal perimeter: ___ ___ cm

**THERAPEUTIC ADJUSTMENT**

**Time of the last take**

**Aspirine __ __ h : __ __ min**

**Clopidogrel __ __ h : __ __ min**

**AVK __ __ h : __ __ min**

**other traitements :** ……………………………………………………………………………………………………………………

**Shour time:** **___ ___**

**Sampling time:** **___ ___**

**FOOD SURVEY**

| **Breakfast** | **Lunch** | **Dinner** |
| --- | --- | --- |
| **_________________ __ __ ___**  **_ __ __ ___________________**  **__ __ __ __________________**  **__ __ __ __________________**  **__ __ __ ____ __ __ ________**  **__ __ __ ____ __ __ ________**  **__ __ __ ____ __ __ ________**  **_________________ __ __ ___**  **_ __ __ ___________________**  **__ __ __ __________________**  **__ __ __ __________________**  **__ __ __ ____ __ __ _____** | **_________________ __ __ ___**  **_ __ __ ___________________**  **__ __ __ __________________**  **__ __ __ __________________**  **__ __ __ ____ __ __ ________**  **__ __ __ ____ __ __ ________**  **__ __ __ ____ __ __ ________**  **_________________ __ __ ___**  **_ __ __ ___________________**  **__ __ __ __________________**  **__ __ __ __________________**  **__ __ __ ____ __ __ ________** | **_________________ __ __ _______________________**  **_ __ __ _______________________**  **__ __ __ ______________________**  **__ __ __ ____________________**  **__ __ __ ____ __ __ ______________________**  **__ __ __ ____ __ __ _____________________**  **__ __ __ ____ __ __ _____** |

|  |  | | 1,2,3 times/ month | | | | | 1,2,3,4,5,6,7 times/ week | | | | | | | | | | | | |
| --- | --- | --- | --- | --- | --- | --- | --- | --- | --- | --- | --- | --- | --- | --- | --- | --- | --- | --- | --- | --- |
|  | never | | **1** | | **2** | **3** | | **1** | | **2** | **3** | | | **4** | | **5** | | **6** | | **7** |
| How much do you consume**?** |  | |  | |  |  | |  | |  |  | | |  | |  | |  | |  |
| • olives / olive oils • Soft drinks • fries • toast with butter • fruits (fresh / compotes / squeezed) • Cakes: modern / traditional • Carrots • Honey jam • Dates • Cucumber • Boiled egg • Brika • Spinach • Lettuce • Green cabbage • Cauliflower • Broccoli • Beans • Beets • Lawyers • offal • Liver • Grapefruit • Marshmallow |  | |  | |  |  | |  | |  |  | | |  | |  | |  | |  |
| How much time do you spend on? |  | |  | |  |  | |  | |  |  | | |  | |  | |  | |  |
| walking ride a bicycle gardening / DIY Housework Sport (swimming, football, aerobics, ..) Sit in front of the TV Sit in front of the computer |  | |  | |  |  | |  | |  |  | | |  | |  | |  | |  |
|  |  | |  | |  |  | |  | |  |  | | |  | |  | |  | |  |
|  |  | |  | |  |  | |  | |  |  | | |  | |  | |  | |  |
|  |  | |  | |  |  | |  | |  |  | | |  | |  | |  | |  |
|  |  | |  | |  |  | |  | |  |  | | |  | |  | |  | |  |
|  |  | |  | |  |  | |  | |  |  | | |  | |  | |  | |  |
|  |  | |  | |  |  | |  | |  |  | | |  | |  | |  | |  |
| How are you sleeping? | |  | | | | | | | | | | | | | | | | | | |
| daily sleep time | | | | .............. H.............. mn | | | | |  | | | |  | |  | | | |  | |
| • daily wake up time | | | | ............... H.............. mn | | | | |  | | | |  | |  | | | |  | |
| • daily nap time Do you feel excessively sleepy during the day? | | | | .............. H.............. mn | | | | |  | | | |  | |  | | | |  | |
| Do you feel excessively sleepy during the day? | | | | never | | | Rarely   | | | | | Regularly | | | | | Often   | | | |
