# Supplementary material for: Effects of Ramadan fasting on aspirin resistance in type 2 diabetic patients
Source: PLoS One. 2018 Mar 12;13(3):e0192590. doi: 10.1371/journal.pone.0192590 (PMC5846719; doi:10.1371/journal.pone.0192590)
Supplement: S2 File — Consent of the patient. (DOC) [file pone.0192590.s002.doc]

**تأثيرات صيام رمضان على وظيفة صفائح الدم**

المستشفى الجامعي فطّومة بورقيبة بالمنستير

قسم الإستعجالي

الهاتف : 73 106 000

السيّد(ة)...................................................................................................................................

لقد تم استدعاؤك إلى قسم الإستعجالي قصد المشاركة في دراسة حول تأثيرات الأدوية الجنيسة (médicament générique : Enoxamed®) على تخثر الدم (agrégation plaquettaire) مع العلم أنك تعاني من أوجاع صدرية (syndrome coronarien aigu).

لذلك نحن بحاجة لأخذ عيّنات من دمك قصد مراقبة تطوّر وضعيّتك الصحيّة, علما و أنّ دمك سيقع حفظه دون ذكر الهويّة في مركز الموارد البيولوجية بالمستشفى الجامعي فطّومة بورقيبة بالمنستير ليقع تحليله بعد ذلك في مختبر مختصّ. لذلك يجب الإشارة إلى أنّ لك الحقّ أن ترفض ذلك في أيّ وقت إذا رأيت أن لا فائدة من العلاج.

التّاريخ : .../.../...

اسم و لقب و إمضاء المريض :.........................................

اسم و لقب و إمضاء الطّبيب :........................................
