# Supplementary material for: Effects of Ramadan fasting on aspirin resistance in type 2 diabetic patients
Source: PLoS One. 2018 Mar 12;13(3):e0192590. doi: 10.1371/journal.pone.0192590 (PMC5846719; doi:10.1371/journal.pone.0192590)
Supplement: S3 File — CONSORT 2010 Flow Diagram (1).doc. (DOC) [file pone.0192590.s003.doc]

**
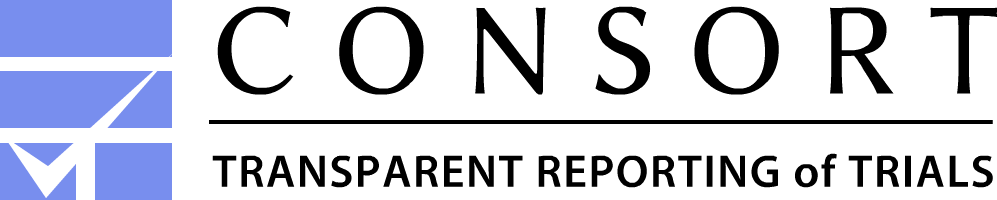
**

**CONSORT 2010 Flow Diagram**

**Analysis**

**Enrollment**

Assessed for eligibility (n= 517 )

Excluded (n= 288 )

♦  Not meeting inclusion criteria (n= 206 )

♦  Declined to participate (n= 42 )

♦  Other reasons (n= 40 )

Analysed (n= 177 )

EXCLUDED (n=52)

Incomplete data at following up (n=19)

Fasting interruption (n=15)

Non compliance with aspirin (n=18)

Randomized (n= 229 )
